# Demographic, socioeconomic, and biological correlates of hypertension in an adult population: evidence from the Bangladesh demographic and health survey 2017–18

**DOI:** 10.1186/s12889-021-11234-5

**Published:** 2021-06-26

**Authors:** Afrin Iqbal, Karar Zunaid Ahsan, Kanta Jamil, M. Moinuddin Haider, Shusmita Hossain Khan, Nitai Chakraborty, Peter Kim Streatfield

**Affiliations:** 1 Maternal and Child Health Division, icddr,b, Dhaka, Bangladesh; 2 Department of Maternal and Child Health, Gillings School of Global Public Health, University of North Carolina at Chapel Hill, Chapel Hill, USA; 3 IAP, World Services, Alexandria, Virginia, USA; 4 Health Systems and Population Studies Division, icddr,b, Dhaka, Bangladesh; 5 Data for Impact, University of North Carolina at Chapel Hill, Chapel Hill, USA; 6 Department of Statistics, University of Dhaka, Dhaka, Bangladesh

**Keywords:** Noncommunicable diseases, Hypertension, Blood pressure, Awareness, Demographic health survey

## Abstract

**Background:**

Bangladesh is well advanced in the epidemiologic transition from communicable to noncommunicable diseases, which now account for two out of three deaths annually. This paper examines the latest nationally representative hypertension prevalence estimates, awareness, treatment, and control—to identify their association with potential correlates.

**Methods:**

The analyses are based on the recent Bangladesh Demographic and Health Survey 2017–18 data. Univariate analyses and bivariate analyses between the outcome variables and individual covariates were carried out. Then chi-square tests were done to see the proportional differences between them. To examine the demographic, socioeconomic and biological factors affecting hypertension, awareness, treatment and control, we used multivariate logistic regression models.

**Results:**

We found that prevalence of hypertension for females and males together aged 35 or more has risen by half between 2011 (25.7%) to 2017 (39.4%). With the broader age range used in 2017, the prevalence is now 27.5% in the population aged 18 years or more. The factors associated with hypertension included older age, being female, urban residence, higher wealth status, minimal education, higher body mass index and high blood glucose level. Following multivariate analyses, many of these characteristics were no longer significant, leaving only age, being female, nutritional status and elevated blood glucose level as important determinants. Over half (58%) of females and males who were found to be hypertensive were not aware they had the condition. Only one in eight (13%) had the condition under control.

**Conclusion:**

In the coming years, a rising trend in hypertension in Bangladeshi adults is expected due to demographic transition towards older age groups and increase in overweight and obesity among the population of Bangladesh. With more women being hypertensive than men, a targeted approach catering to high risk groups should be thoroughly implemented following the Multisectoral NCD Action Plan 2018–2025. Acting in close collaboration with other ministries/relevant sectors to bring an enabling environment for the citizens to adopt healthy lifestyle choices is a prerequisite for adequate prevention. While screening the adult population is essential, the public sector cannot possibly manage the ever-expanding numbers of hypertensives. The private sector and NGOs need to be drawn into the program to assist.

**Supplementary Information:**

The online version contains supplementary material available at 10.1186/s12889-021-11234-5.

## Introduction

Hypertension or elevated blood pressure (BP), one of the major non-communicable diseases (NCDs), is a leading health risk globally [1] and the major risk factor for preventable premature deaths worldwide [2]. In 2010, approximately one-third of the world’s adults; aged 20 years or older; had hypertension. In the decade 2000 to 2010, there was a small decrease (2.6%) in age-standardized prevalence of hypertension in high-income countries, however, in contrast, prevalence increased by 7.7% in low-and middle-income countries [3]. If left uncontrolled, hypertension may lead to complications including cardiovascular and cerebrovascular diseases, peripheral vascular diseases, renal failure, visual impairment, dementia etc. [4]. In a 2015 study using global burden of disease data, over 19% of all deaths were linked to elevated Systolic Blood Pressure (SBP) (> 140 mmHg) leading to cardiovascular deaths (4.7 million) and cerebrovascular deaths (2.6 million) [5]. Countries of lower developmental status saw greater increases in the number of deaths linked to elevated BP than most developed countries [6].

The burden of NCDs on both morbidity and mortality has been recognized for decades [7] . The rising prevalence of hypertension did not necessarily raise awareness regarding the condition, or increase care seeking, the result being many cases of uncontrolled elevated BP. Studies show that an increase in awareness campaigns regarding the presence of the condition, lifestyle modifications, early care-seeking and treatment can help in lowering elevated BP to normal levels. However, a recent Lancet article reported on data from 123 national health examination surveys in high-income countries and demonstrated that there is wide variation in levels of awareness, of availing treatment and of getting BP under control across those countries [8]. Compared to low- and middle-income countries in 2010, high-income countries had almost double the levels of awareness and treatment and four times the level of control among patients with hypertension. Comparing the trend from 2000 to 2010, the awareness regarding hypertension, being treated and having BP under control increased substantially in high-income countries. While those in low- or middle-income countries reported smaller increases in awareness, and in treatment, and a slight decrease in control [3].

The national prevalence levels of hypertension in the STEPwise approach to surveillance (STEPS) surveys among various countries in South Asia are alarming, for example, Sri Lanka (26% in 2015); Nepal (25% in 2019); Bhutan (26% in 2014); Myanmar (26% in 2014) [9]. The different STEPS surveys in South Asia may not be comparable with each other due to different age range of respondents and different time periods, however, an overview by the World Health Organization (WHO) stated that a third of the population in South East Asia are hypertensive [10]. Two rounds of Bangladesh Demographic and Health Surveys (BDHS) in 2011 and 2017–18 also show a significant increase in hypertension prevalence among males and females 35 years and over from 19.4 to 24.0% and 31.9 to 44.6%, respectively [11, 12]. Such a rapid rise in hypertension prevalence between surveys warrants an examination of the patterns and determinants of hypertension and the need to identify its association with potential demographic, socioeconomic and biological predictors in Bangladesh. The DHS reports are descriptive and contains no multivariate analyses, but the data provide the potential for deeper understanding of the patterns and determinants of the outcome variable (in this case, hypertension).

More women than men tend to be hypertensive in Bangladesh and 57% of the females in Bangladesh are within the reproductive age group [13]. The Bangladesh Maternal Mortality and Health Care Surveys (BMMS) showed that between 2010 and 2016 the proportion of deaths to women of reproductive age due to circulatory diseases rose by almost half, from 16 to 23% [14, 15]. This rise may be in line with the rise in prevalence of hypertension seen between BDHS 2011 and BDHS 2017–18 [11, 12] . For women during pregnancy, the existence of hypertension is a serious risk factor for eclampsia, which as a cause of maternal deaths, has also risen, from 20% in BMMS 2010 to 24% in BMMS 2016 [14, 15].

With ageing populations and rising trends in hypertension prevalence, there is clearly a huge burden of mortality and morbidity due to hypertensive complications in this region. The BDHS 2011 found that more than half of the adult population aged 35 years or more were not aware of their hypertensive status, and two-thirds of those who were aware and taking medication, did not have their condition under control [11]. Six years later, the most recent BDHS showed a similar pattern among the adult population aged 18 years and more regarding awareness, treatment and getting the condition under control [12]. Another nationally representative survey, STEPS 2018, reported approximately half of the hypertensives to be unaware of their condition and only 14% having the condition under control [16].

Considering the huge burden of NCDs, the Government response has been to develop a long list of policy and strategy documents, many based on the six objectives of the WHO’s 2013–2020 Action Plan for the Global Strategy for the Prevention and Control of NCDs. A recent review of 51 such documents [17] has concluded that while the establishment of NCD corners at 300 primary level facilities at Sub-district level (Upazila Health Complexes) has been an effective way to provide screening and treatment, other robust steps are required to implement timely interventions. A major gap is that Bangladesh still lacks any NCD focused national surveillance program at community or facility level, and only a few tertiary hospitals maintain such a system [18].

This paper examines the latest BDHS 2017–18 data for nationally representative estimates of prevalence of hypertension, of being aware, of being treated and of having the condition under control and to identify their association with potential correlates such as age, sex, place of residence, educational achievement, socioeconomic status, nutritional status and coexistence of elevated blood sugar. Based on the findings, this paper also discusses possible future directions for the country to achieve its intended goals.

## Methods

**Fig. 1:**
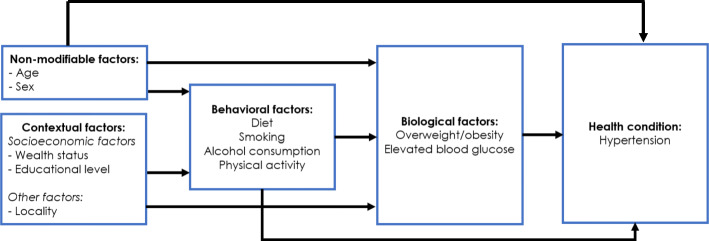
Conceptual Framework

This paper uses data from the BDHS 2017–18. To examine the latest nationally-representative estimates of hypertension prevalence, awareness, and management among the adult population in Bangladesh and to identify its association with potential predictors, we followed an adaptation of the conceptual framework proposed by Wong and colleagues in 2005 (Fig. 1) [19].

The primary dependent variable of the study was hypertensive status among adults, and the independent variables that comprised the non-modifiable, contextual, and biological risk factors were based on the data collected under BDHS 2017–18. Age and sex were considered as non-modificable factors that affect hypertension, whereas household wealth, educational attainment and place of residence (urban/rural) were considered as contextual factors. For the analysis of biological risk factors, overweight/obesity and elevated blood glucose level were considered. Following this conceptual framework, we hypothesized that the aforementioned non-modificable, contextual, and biological factors affect all four dimensions of hypertension (i.e., prevalence, awareness, treatment, and control) [20]. A weakness of this famework is the absence of certain behavioral factors, such as the limited mobility of females outside the home, at least compared to males. There are obvious negative implications for female physical activity which is a risk factor for hypertension. Data on physical activity was not collected in the BDHS surveys. Data on diet, tobacco and alcohol consumption were also not collected in the survey.

### Study population, sampling, data source and modalities

This study utilized nationally representative survey data conducted in 2017–18. The 2017–18 BDHS is the eighth national survey which reported the demographic and health status of women and children from a nationally representative sample, with selected information on men from the same households. In total, 20,250 households were surveyed following a two-stage stratified sampling procedure from 675 clusters in the first stage. In the second stage, a systematic sample of 30 households was selected per enumeration unit. Details of the survey methodology can be found elsewhere [12].

The 2017–18 BDHS included biomarker measurements for the adult population. Blood pressure and blood glucose tests were done on all adults age 18 and over in one fourth of the sampled households. For BP measurement, LIFE SOURCE® UA-767 Plus BP monitors were used. Following the WHO guideline, three measurements of blood pressure were taken in a seated position by trained technicians at approximately 10 min intervals between each measurement. The average of the last two measurements was used to report respondent’s blood pressure values. To determine elevated blood glucose level, fasting blood glucose (FBG) levels were measured using a HemoCue 201+ glucometer after at least 8 h of overnight fasting.

BDHS 2017–18 also included height and weight measurements of all adults eligible for BP and blood glucose testing. Weight measurements were obtained using lightweight, electronic SECA 878 scales with a digital screen. Measuring boards made by Shorr Productions were used to carry out height measurements. BMI was calculated by dividing the respondents weight measured in kilograms by square of height measured in meters.

For this analysis, we selected the respondents who gave their full consent to do both biomarker and anthropometric measurements and excluded pregnant women, women giving birth in the preceding 2 months and if any case found with missing observations for all the variables used in this study; consequently, the final study sample size was 12,769 (7248 women and 5521 men).

### Outcome variables

The outcome variables are prevalence of hypertension (hypertensive); awareness of the respondent regarding their own hypertensive state (aware); treatment status of hypertensive respondents (treated); and level of blood pressure control (controlled).

The 2017–18 BDHS defined hypertension following the 2003 American Heart Association (AHA) guidelines for cut-off points for blood pressure measurements [21]—respondents were identified as ‘hypertensive’ if their systolic blood pressure values were 140 mmHg or higher and/or diastolic blood pressure values were 90 mmHg or higher on the day of the survey, or they were currently taking any medication for lowering blood pressure. New guidelines were issued by the AHA in 2017, but these were too late for the fieldwork of the BDHS 2017–18 [22]. The threshold for BP cut-offs in 2003 AHA guideline is in line with other widely used guidelines (European Society of Cardiology/European Society of Hypertension Arterial Hypertension Guidelines (ESC/ ESH), International Society of Hypertension Global Hypertension Practice Guidelines (ISH) etc.)

‘Aware’ was defined as the hypertensive respondents being aware that they have elevated blood pressure, or hypertension, conveyed to them by a physician or any health care provider. ‘Treated’ was defined as the hypertensive respondents who reported that they were currently taking any antihypertensive medication on the day of the survey. ‘Controlled’ was defined as the hypertensive respondents who had BP below the threshold of 140 mmHg systolic and 90 mmHg diastolic BP while taking antihypertensive medication on the day of the survey.

### Explanatory variables

While selecting the explanatory variables, we primarily used a conceptual framework, a review of relevant literature for Bangladesh, and availability of data in BDHS. We considered a number of factors which could be associated with the outcome variables. These include- *demographic factors*—age and sex of the respondent, *socio economic factors*—household wealth quintile, educational status, place of residence and *biological factors*—Body Mass Index (BMI), and elevated blood sugar level.

Male and female adult’s age was self-reported. The age of the respondents was divided into six categories. The household wealth quintile was divided based on household wealth index created using principal component analysis of household assets for both urban and rural areas [12]. Self-reported level of educational attainment was divided into six different categories. Place of residence was categorized into two—respondents residing in urban or rural areas. BMI was categorized into three groups (underweight, normal and overweight or obese) and BMI over 25 kg/meter^2^ was considered as overweight/obese. Elevated blood sugar level (> 7 mmol/L) was a binary variable where the respondent either had elevated blood sugar or did not.

### Statistical analyses

Univariate analyses of the explanatory variables were performed. Later, bivariate analyses between the outcome variables and individual covariates were carried out followed by chi-square test to see the proportional differences between them.

To examine the demographic, socioeconomic and biological factors affecting hypertension, awareness, treatment and control, a multivariate logistic regression model was used with the following regression specification:

$$ logit\left({Y}_i\right)=\ln \left(\frac{\pi }{1-\pi}\right)=\alpha +{\beta}_i{X}_i+{\gamma}_i{S}_i+{\theta}_i{BMI}_i+\varphi Elevated\ {BG}_i+{\varepsilon}_i $$

where Y, the outcome variable, is a dichotomous variable with the value equal to 1 if the individual *i* is hypertensive or aware or treated or controlled, (0 otherwise); *π* is the probability of being hypertensive, aware, treated and controlled for individual *i* (i.e. *Y*_*i*_ = 1); *X*_*i*_ is a vector of explanatory demographic variables such as sex (1 if female, 0 if male) and age of individual *i* (divided into six categories); *S*_*i*_ is a vector of explanatory socioeconomic variables such as place of residence (1 if rural, 0 if urban), household wealth quintiles (five categories), and educational attainment of individual *i* (four categories); *BMI*_*i*_ is the body mass index of individual *i* (three categories); and *Elevated BG*_*i*_ is elevated blood glucose of individual *i* (three categories).

Both crude and adjusted odds ratios were calculated for each covariate at 95% level of confidence. Appropriate sampling weights for BDHS 2017–18 were applied by using Stata’s survey estimation procedures (“svy” command) to get nationally representative estimates after adjusting for sample clusters [23].

Following the above model specification, factors affecting hypertension prevalence, awareness, treatment, and control were examined by using separate multivariate logistic regression models. We used confounder-adjusted logistic regression models to examine the relation between the explanatory variables and the predicted probability of having hypertension among sampled respondents aged 18 years or above, who were representative of the adult population of Bangladesh. The predicted probability of an explanatory variable was estimated by calculating the mean sample probability of having hypertension constant at the mean value of each variable, so that we can interpret it as the probability that an otherwise average respondent would have hypertension. We compared the predicted probabilities from different groups to identify the most at-risk population groups.

## Results

Among the study participants, 56.8% were female and 43.2% male. Approximately 21% of respondents were 55 years or more, with more than three quarters residing in rural areas. Distribution of wealth index was similar between men and women. More males had college or higher education than females, while more than a quarter of the females were uneducated. More females were overweight or obese; 1.7 times higher than their male counterpart. Approximately 9% of the participants had elevated blood glucose level and more males had not checked their blood glucose compared to females (Table 1).

**Table 1:** Background characteristics of the study participants

	All % (number)	Male % (number)	Female % (number)
**Age (in years)**			
18–24	20.3 (2590)	15.9 (879)	23.6 (1712)
25–34	24.9 (3175)	21.9 (1214)	27.1 (1961)
35–44	20.2 (2573)	21.6 (1190)	19.1 (1383)
45–54	14.1 (1796)	15.5 (856)	12.9 (939)
55–64	11.3 (1439)	12.7 (700)	10.2 (738)
65+	9.4 (1196)	12.4 (682)	7.1 (515)
**Place of residence**			
Urban	27.5 (3509)	28.2 (1559)	26.9 (1950)
Rural	72.5 (9260)	71.8 (3962)	73.1 (5298)
**Wealth index**			
Poorest	19.0 (2430)	18.4 (1014)	19.5 (1416)
Poorer	19.5 (2493)	19.6 (1079)	19.5 (1414)
Middle	20.4 (2606)	20.8 (1150)	20.1 (1456)
Richer	19.8 (2527)	20.2 (1113)	19.5 (1414)
Richest	21.3 (2713)	21. (1165)	21.4 (1548)
**Educational attainment**			
No education	25.4 (3238)	23.2 (1282)	26.9 (1956)
Primary	29.9 (3826)	31.3 (1727)	28.9 (2099)
Secondary	25.9 (3308)	22.4 (1239)	28.6 (2069)
College or higher	18.8 (2397)	23.1 (1273)	15.5 (1124)
**Body mass index**			
Underweight	17.1 (2183)	19.8 (1093)	15.0 (1090)
Normal	58.6 (7481)	62.7 (3459)	55.5 (4022)
Overweight and Obese	24.3 (3105)	17.6 (969)	29.5 (2136)
**Elevated blood glucose**			
No	84.4 (10,777)	83.1 (4587)	85.4 (6190)
Yes	9.3 (1182)	9.7 (537)	8.9 (645)
Not tested	6.3 (810)	7.2 (397)	5.7 (413)
**Total**	**12,769**	**5521**	**7248**

The levels of hypertension in the 2017–18 BDHS are remarkably high, with 27.5% among adults of 18 years or more. Comparison with the earlier survey, BDHS 2011 using ages 35 years or more, shows a substantial rise in prevalence between 2011 and 2017–18 from 26 to 39%.

The bivariate analysis shows that the prevalence of hypertension rises steadily with age to a six-fold higher level among those aged 65+ compared to those under 18–24 years (Table 2). The prevalence of being aware, treated and having the condition under control also rises with age, however a slight drop in the oldest age group is observed.

**Table 2:** Distribution of prevalence of hypertension, awareness, treatment and condition controlled, by explanatory variables

Explanatory variables	Hypertensive (***N*** = 12,769)		Aware (***N*** = 3479)		Treated (N = 3479)		Controlled (N = 3479)	
	Percent	χ^2^ test *p*-value	Percent	χ^2^ test p-value	Percent	χ^2^ test p-value	Percent	χ^2^ test p-value
**Age (in years)**								
18–24	8.5	< 0.001	17.5	< 0.001	12.3	< 0.001	10.3	0.108
25–34	16.2		33.9		26.1		12.5	
35–44	28.5		37.8		32.4		12.8	
45–54	38.5		45.8		41.1		12.8	
55–64	46.7		53.4		48.5		15.9	
65+	54.4		48.0		42.8		9.2	
**Sex**								
Male	26.0	0.004	33.4	< 0.001	28.5	< 0.001	9.0	< 0.001
Female	28.2		48.8		42.9		15.0	
**Place of residence**								
Urban	28.2	0.015	46.8	< 0.001	41.5	< 0.001	14.9	0.009
Rural	26.9		40.7		35.2		11.6	
**Wealth index**								
Poorest	23.5	< 0.001	32.3	< 0.001	27.5	< 0.001	8.9	0.001
Poorer	24.8		36.5		30.3		10.1	
Middle	26.5		41.7		37.4		12.0	
Richer	28.0		46.2		39.9		15.1	
Richest	32.9		50.7		45.0		14.9	
**Educational attainment**								
No education	35.4	< 0.001	43.2	0.120	38.9	0.159	10.6	0.050
Primary	26.9		44.2		37.8		13.5	
Secondary incomplete	22.7		39.9		35.5		13.2	
Secondary complete or higher	23.2		41.2		33.6		13.8	
**Body mass index**								
Underweight	17.7	< 0.001	31.1	< 0.001	26.2	< 0.001	8.4	0.006
Normal	23.9		40.0		35.2		11.8	
Overweight/Obese	42.2		49.1		42.6		14.7	
**Elevated blood glucose**								
No	25.4	< 0.001	39.7	< 0.001	34.1	< 0.001	11.9	0.043
Yes	45.5		57.3		52.9		14.6	
Not tested	25.6		40.6		34.0		15.2	
**TOTAL (95% CI)**	**27.3 (26.3–28.3)**		**42.5 (40.5–44.5)**		**36.9 (35.1–38.9)**		**12.5 (11.3–13.8)**	

Note 1: When chi-square tests were performed to see the proportional differences between the outcome variables and explanatory variables, they were significant except- education and aware; education and treated, age category and controlled

Note 2: The relatively high non-response rate among the study respondents (any males and females aged 18 years or more) was random in nature and did not distort the outcome variable estimates

Prevalence of hypertension was found to be similar in urban and rural populations. Also, females tend to be slightly more hypertensive than men, but have substantially higher levels of awareness, taking medications, and being in control of their high blood pressure levels.

The richest quintile has more than one third higher prevalence than the poorest with a steady rise in prevalence with increasing wealth. Those with no education have the highest prevalence of hypertension and the lowest level of awareness.

The prevalence of being hypertensive, aware, treated and controlled in the overweight or obese category is significantly higher than those underweight or normal. This rising gradient with BMI or weight is consistent with that of economic status, but not with that of education.

We observed a relatively high non-response rate among the study respondents (any males and females aged 18 years or more). During our exploration, we found that this non-response pattern was quite random in nature and the little differences are unlikely to affect the outcome variable estimates.

**Fig. 2:**
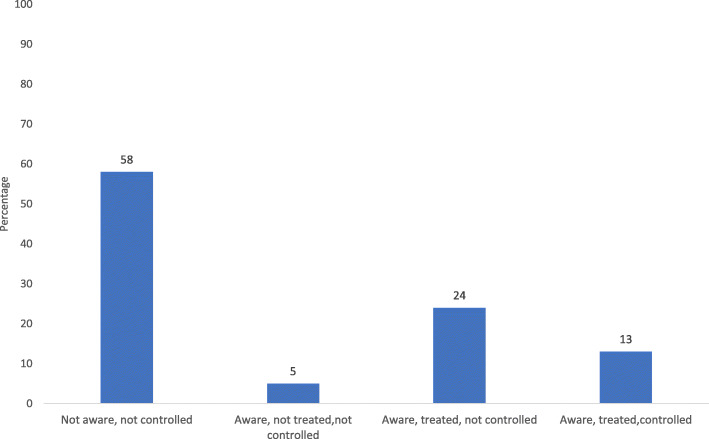
Level of awareness, treatment and control among hypertensive adult population of 18 years or more

Among those respondents aged 18 years or more who were hypertensive, more than half (58%) of them were not aware that they had the condition (Fig. 2). Approximately a quarter (24%) of the hypertensive adults were aware that they had the condition, were taking treatment, but did not have the condition under control. Only 13% were aware, were taking treatment, and had the condition under control.

When hypertension prevalence, awareness, treatment, and control were examined in a logistic regression, the patterns were as follows (Table 3). Considering the 35–44-year age group as the reference category, the likelihood of being hypertensive increased gradually with age, and the oldest group had 4.5 times higher odds of being hypertensive than the reference group, after adjusting for the relevant covariates. This is consistent with the bivariate analysis. For awareness, there is also a rising significant gradient with age, though at smaller magnitude than for prevalence. For treatment there is a significant rising gradient with age. For control, the younger group are less likely to have their condition under control than the older (55–64 year) age group, though the oldest group (65+) has a low level of control.

**Table 3:** Adjusted odds ratios (AORs) for being hypertensive, aware, treated and controlled by explanatory variables

	Adjusted Odds Ratios (95% Confidence Interval)			
Explanatory variables	Hypertensive	Aware	Treated	Controlled
**Age (in years)**				
18–24	0.26 (0.22–0.32)**	0.39 (0.25–0.60)**	0.33 (0.20–0.54)**	0.77 (0.44–1.34)
25–34	0.47 (0.40–0.54)**	0.80 (0.61–1.06)	0.70 (0.52–0.95)*	0.88 (0.59–1.30)
35–44	(ref)	(ref)	(ref)	(ref)
45–54	1.74 (1.48–2.05) **	1.49 (1.16–1.93)**	1.55 (1.19–2.02)**	1.04 (0.74–1.46)
55–64	2.85 (2.46–3.30) **	2.47 (1.94–3.14)**	2.51 (1.96–3.22)**	1.57 (1.13–2.19)*
65+	4.52 (3.82–5.35) **	2.38 (1.84–3.09)**	2.37 (1.81–3.11)**	0.93 (0.64–1.37)
**Sex**				
Male	(ref)	(ref)	(ref)	(ref)
Female	1.32 (1.20–1.46)**	2.30 (1.94–2.73)**	2.25 (1.89–2.68)**	2.01 (1.56–2.58)**
**Place of residence**				
Urban	(ref)	(ref)	(ref)	(ref)
Rural	1.00 (0.88–1.13)	0.94 (0.77–1.14)	0.90 (0.75–1.09)	0.88 (0.68–1.14)
**Wealth index**				
Poorest	(ref)	(ref)	(ref)	(ref)
Poorer	1.03 (0.86–1.22)	1.29 (0.97–1.72)^†^	1.23 (0.92–1.64)	1.15 (0.76–1.76)
Middle	1.03 (0.88–1.22)	1.49 (1.12–1.98)*	1.57 (1.19–2.07)*	1.33 (0.85–2.09)
Richer	1.08 (0.90–1.29)	1.77 (1.32–2.37)**	1.73 (1.31–2.30)**	1.63 (1.09–2.45)*
Richest	1.10 (0.91–1.32)	1.81 (1.32–2.46)**	1.83 (1.35–2.47)**	1.43 (0.93–2.18)
**Educational attainment**				
No education	(ref)	(ref)	(ref)	(ref)
Primary	1.08 (0.95–1.23)	1.32 (1.08–1.61)*	1.21 (0.98–1.48)^†^	1.42 (1.07–1.90)*
Secondary incomplete	1.15 (0.99–1.34)^†^	1.18 (0.92–1.51)	1.20 (0.93–1.55)	1.38 (0.97–1.97)^†^
Secondary complete or higher	1.17 (0.99–1.40)^†^	1.42 (1.06–1.91)*	1.25 (0.92–1.68)	1.65 (1.12–2.43)*
**Body mass index**				
Underweight	(ref)	(ref)	(ref)	(ref)
Normal	1.82 (1.60–2.07)**	1.52 (1.18–1.96)*	1.58 (1.20–2.07)*	1.27 (0.85–1.90)
Overweight and Obese	4.37 (3.70–5.16)**	1.99 (1.50–2.63)**	1.96 (1.45–2.64)**	1.31 (0.86–1.99)
**Elevated blood glucose**				
No	(ref)	(ref)	(ref)	(ref)
Yes	1.52 (1.31–1.78)**	1.58 (1.27–1.96)**	1.67 (1.36–2.08)**	1.09 (0.82–1.46)
Not tested	0.90 (0.74–1.10)	0.97 (0.70–1.34)	0.94 (0.66–1.34)	1.24 (0.80–1.94)
Constant	0.12 (0.09–0.15)**	0.11 (0.07–0.17)**	0.09 (0.06–0.14)**	0. 1.24 (0.02–0.08)**
**Total**	**12,769**	**3479**	**3479**	**3479**

**p < 0.001, * *p* < 0.05, ^†^ *p* < 0.10

Sex was also found to be a significantly associated with hypertension—compared to men, women have 32% higher odds to be hypertensive, after adjusting for the relevant covariates. Odds of being aware of hypertensive status, taking medications, and controlling blood pressure level are also found to be significantly higher for females.

Urban-rural residenceand education all lost their significance for hypertension prevalence when adjusted for other explanatory variables. For hypertension, wealth index also lost significance when adjusted for other variables, however, for awareness and treatment, the rising trend with wealth persists, even though it was not statistically significant for prevalence, itis significant for the top three wealth quintiles.

Nutritional status retained its significance after being adjusted for other explanatory variables. BMI is associated with significantly higher prevalence for normal and overweight or obese respondents than for those underweight. They were also more likely to be aware and to get treatment, but not to control their condition.

Individuals with primary or secondary complete or more education are significantly more likely to be aware and to be in control of their condition, though not for treatment.

Finally, those respondents who had elevated blood glucose were significantly more likely to have hypertension, to be aware of their condition, and to get treatment, though like BMI they were not more likely to have the condition under control.

We also estimated predicted probabilities to understand the overall hypertensive prevalence by selected explanatory variables (Additional file 1). Keeping all other variables constant at their means, the probability of being hypertensive rises steadily from 9% for the youngest category [16–22] to 60% for the oldest category (65+), with all of the age categories significantly different from each other. Females have a significantly higher probability of being hypertensive. Being of normal BMI or overweight or obese brings significantly higher risk of hypertension than being underweight. Also being diabetic increases the probability of being hypertensive. As with AORs, place of residence, wealth index and education are not significant.

## Discussion

Prevalence of hypertension among adults age 35 and over increased substantially between BDHS 2011 and BDHS 2017–18. Among adults age 35 years or above, 32% (95% CI: 29.7–33.3%) women and 19% (95% CI: 17.6–20.7%) men were found to be hypertensive, whereas it increased to 44.6% (95% CI: 42.8–46.4%) women and 34% (95% CI: 32.1–36%) men in 2017–18 [11, 12]. It is unusual for a chronic condition to change rapidly, but we are confident that the data are reliable across the two surveys. The same DHS global questionnaire was used, the data collection company was unchanged, measurement instruments and training were the same. The age structure changed less than 0.5 percentage points. A level of 110 mmHg SBP is often considered a threshold of increased risk of cardiovascular or cerebrovascular events [5]. We examined the distributions of both SBP and DBP for comparable samples between 2011 and 2017–18 and found that the upward change in hypertension prevalence in Bangladesh has involved a shift in the entire distributions of SBP and DBP to the right. This shift has particularly occurred among those below the hypertension thresholds of 140 mmHg for SBP and 90 mmHg for DBP (Additional file 2). This aligns with the findings from other South Asian countries [24]. A similar trend in rise in hypertension could be seen in the NCD risk factor survey in 2010 and the national STEPS survey in 2018 among adults of 25–69 years (17.9% vs. 25%) [16, 25]. For a comparable age range (40–69 years, both sexes), hypertension prevalence estimated in BDHS 2017–18 and national STEPS 2018 were found to be similar [12, 16].

Increasing age is a powerful determinant of hypertension, which is consistent with the Hirai study of 10 countries which had included BP measurements using DHS data, also with non-DHS countries like Germany [26, 27].

This study showed adult females have higher prevalence of hypertension than males. We observed the same in 2011 among adults age 35 years or over as well, where one-in-three (32%) women and one-in-five (19%) men were found to be hypertensive [11]. Higher female prevalence is less common than male in other DHS surveys [27, 28] . This may be due to very different age ranges surveyed in different countries, many focusing only on the reproductive ages (15–49 years) for females, but up to 59 or older for males. In a systematic review of 53 (non DHS) hypertension studies in Bangladesh the sex differential was presented in 25, of which in 17 studies, females had higher prevalence [29]. While exploration on why hypertension among females in Bangladesh found to be higher than males is necessary, usage of oral contraceptive pills (OCPs) may have played a role. BDHS 2017–18 showed 25% of women of reproductive age use OCPs with a peak around 30–34 year age group [12]. As we do not have the duration of OCP usage, it is difficult to draw any association between use of OCPs and being hypertensive from this survey. However, studies have shown that use of OCPs can be associated in a small number of cases with hypertension and related complications [30, 31].

Apart from age and sex, the strongest differential is nutritional status, where being overweight or obese carries more than four times the risk of hypertension than being underweight. While looking at other studies, we found that though age groups were not comparable, the 2010 national NCD Risk Factor survey in Bangladesh reported prevalence of overweight and obesity together to be higher in women (33.7%) than in men (18.3%) which increased during the 2018 National STEPS survey (21.6% in women vs 13% in men) [16, 25]. Similarly, in the BDHS 2011 and 2017–18, study participants aged 35 years or more showed a higher proportion of overweight/obese from 26.6% in 2011 rising to 40.7% in 2017–18 in women and in men from 16.0% in 2011 rising to 28.7% in 2017–18 [11, 12]. The higher proportion of overweight and obese could partly explain the increase in prevalence of hypertension between BDHS 2011 and BDHS 2017–18 among adults age 35 and over. This is alarming as the association of higher BMI and hypertension is robust, and when south Asian populations are considered, BMI has been found to be associated with several NCDs including hypertension at a much lower threshold level compared to other populations [28, 32–34]. Overweight individuals are more than twice as likely, and obese individuals are more than six times as likely, to suffer from hypertension as individuals with a normal BMI [35]. This finding of a strong positive association between higher BMI and hypertension is consistent with an earlier study among an Indian population and studies from other parts of the world [36–44]. Though there are different schools of thought regarding the BMI cut-off for Asian population, we have however, decided to use the most widely utilized and internationally accepted categories for our analysis. In addition, the Government of Bangladesh also uses this BMI cutoffs for program monitoring and strategic planning purposes. With the nutritional transition of Bangladesh, the steady increase in BMI has grave consequences related to premature NCD related morbidity and mortality. The possible reasons for such a strong association may be due to several genetic and metabolic reasons [34, 45–47] and demand prospective cohort studies to understand such associations in Bangladeshi adults.

The need for lifestyle modification to reduce BMI, improve diet and increase physical activity, especially for females is crucial. In the USA the Blue Zones Project, a multifaceted approach of reducing consumption of red meat and salt, increasing vegetables, fruits, whole grains and nuts consumption, and using residents recruited from women’s organizations in their localities to influence dietary change could be effective for Bangladesh if adapted to the country context [48]. However, it should be kept in mind that no matter which country it is, individual change is easier to be sustain if there are enabling environments. Food systems, marketing patterns, purchasing power, and cultural aspects play an important role in choosing the lifestyle for themselves and their family. An enabling environment requires supportive regulatory, legislative and fiscal policies at national level as well as availability of a supportive environment at school, workplace, community, and availability of healthy choices within normal purchasing power.

It is surprising that in our study the association of place of residence (urban/rural) with prevalence of hypertension was insignificant. In many other studies, hypertension tends to be higher in urban areas, even in Bangladesh [29, 49]. When we looked at socio-economic differentials, our study found that the better off have higher, but not necessarily significantly greater prevalence of hypertension than the lower quintiles. An analysis of the 2011 BDHS found that hypertension prevalence was higher among the rural poor than the rural better off. But the opposite was the case in urban areas, where the better off had higher prevalence [50]. This is also the case in other countries. Among countries which have collected NCD data with the DHS module, Nepal 2016, Egypt 2008, Lesotho 2009, and Namibia 2013 have a rising hypertension trend with higher wealth [51–54]. While Albania 2008/09, Azerbaijan 2006, and Ukraine 2007 have a declining trend with higher wealth [9, 55, 56] .

Where awareness is concerned, our study showed that more females are aware compared to their male counterparts. The 2010 NCD risk Factor Survey and 2018 national STEPS survey also showed more females had ever measured their BP compared to their male counterparts (77.2% females Vs 55.5% males in 2010 and 82.9% females Vs 57% males in 2018) [16, 25]. This could be due to the relatively high antenatal care coverage nationally [57], where many women of reproductive age make multiple antenatal care visits during pregnancy and have their BP measured. We also observed, the better off are likely to be more aware of their condition and being treated for it than less well-off, but when having the situation under control are considered, no pattern is observed. This could be interpreted to mean that the better off group may be more likely to be screened than the less well-off group, but due to the low cost of popular antihypertensive drugs, and over the counter drug availability in Bangladesh, no difference between the odds ratio of treated and controlled group can be seen.

There are two important aspects of raising awareness. The first is that the population needs to be informed that hypertension is often a ‘hidden’ condition, and they need to be checked periodically. This falls under Action area one (Advocacy, leadership and partnerships) of the Multisectoral Action Plan 2018–2025 launched recently by the Bangladesh government’s Non Communicable Disease Control Program: “Raise public and political awareness / understanding about NCDs and their risk factors through social marketing, mass media and responsible media reporting” [58]. The second is the capacity of health facilities to carry out screening. According to the 2017 Bangladesh Health Facility Survey Report, seven out of ten facilities offer hypertension services [59]; but the readiness of facilities to provide quality services was found to be very low.^1^ There was a widespread lack of guidelines, and less than one third of staff had received training in hypertension, but BP measuring equipment was widely available (91%), even at Community Clinics (88%) which each cater to around 6000 rural population. Urban facilities ranked higher in ‘readiness’ than rural in this regard.^2^

As the current Bangladesh target population (viz., adult males and females age 40 yours or older, and pregnant women) for NCD screening exceeds 50 million, this is a huge workload for health workers in the community and facilities. To expand screening capacity rapidly to accommodate this huge target population, one approach would be to add pharmacies which already have capacity for screening, if not for prescribing treatment. As an example, there are 5500 Blue Star pharmacies in rural areas, and 2500 in urban areas, with 1000 more becoming available soon, which have staff trained to measure BP. There are another 150 grade-A pharmacies which could be drawn into the urban network of screening facilities to then refer positive patients to higher level facilities for treatment [60].

Where control is concerned, this study revealed that only 13% of hypertensives had the condition under control at the time of survey. Only 13% of hypertensive patients having had their condition controlled was also reported in a systematic review of several studies in the early 2000’s based on developing countries [61]. While some studies have shown poor medication adherence as the primary reason for uncontrolled hypertension [62], other studies revealed possible systematic, cultural and personal factors behind poor medication adherence or non-adherence, i.e. .- perception of hypertension as a non-severe condition and discontinuing medication when symptoms disappeared or the patient felt better, low income or poor households, general dissatisfaction in healthcare providers and services, alternate medication sources such as- herbal remedies and spiritual healers, etc. [63]. Rigorous exploration is also required to understand poor control among Bangladeshi hypertensive adults.

The experience of the Kaiser Permanente Hypertension program in California was encouraging where this program increased hypertension control from 44% in 2001 to 80% in 2009, and 90% by 2013 [64]. This is partly attributed to allowing follow-up visits to Medical Assistants (MA) in more local or peripheral facilities. These MAs measured BP and when needed, reported back to the primary physician for modification of the treatment plan. This greatly reduced the burden on physicians, facilitated access closer to patients’ homes, and reduced costs to patients and the health system. In the Bangladesh context, we propose a minor change in the existing hypertension screening and treatment guideline [65]. Rather than visiting a physician every month for follow-up and medications as suggested in the national guideline, after the initial visit with a physician for screening and prescription for medication, follow-up visits could be conducted at primary-level public facilities (i.e., at union-level facilities or Community Clinics) for medications and basic physical assessments including BP measurement. For those patients who do not get their condition under control, they may be referred back to the primary physician at the higher-level facilities (i.e. at district hospitals or sub-district health complexes). Thought can be given to how to delegate follow-up visits in urban areas where the health system possesses several other complexities.

In summary, the Bangladesh’s National NCD Program needs to adopt new approaches to ensure a larger proportion of the target population can be screened. Then more investment is needed in surveillance and follow-up to raise the level of compliance or adherence to treatment so that more of the detected hypertensives can successfully get the condition under control.

### Limitations

The blood pressure measurements taken in 2017–18 BDHS were not intended to provide a clinical diagnosis of the condition. It was rather to provide a cross-sectional assessment to prevalence of high blood pressure at the population level at the time of the survey. BDHS does not include home/ automated monitoring for optimal hypertension screening as it may not be feasible in an LMIC setting. The results do not reflect a clinical diagnosis of hypertension, where, generally, a clinical setting is required and an individual’s blood pressure would be taken and monitored over a prolonged period, with relevant history for that individual; i.e.- clinical history, family history, medication, etc. An individual’s blood pressure is taken in the survey for 1 day only, which is recorded to provide information on the national status of high blood pressure, which is an important NCD associated risk factor. Behavioral correlates for hypertension prevalence, awareness, and management were not considered, as those data were not available under BDHS.

Lastly, the data source used for this study did not collect information on ideation and norms in hypertension care-seeking and control, which remains a gap in this analysis. Further research is needed to understand beliefs, practices, and social/cultural norms around noncommunicable diseases such as hypertension. Also, contrary to available evidence, why the difference in hypertension prevalence between urban and rural areas no longer exists required further exploration.

## Conclusion

The rapid rise in hypertension prevalence is a major public health concern for Bangladesh. Achieving the health-related Sustainable Development Goals by 2030 cannot be materialized without addressing major NCDs like hypertension. From the findings of this study, it is clear that the rising trend in hypertension in Bangladeshi adults is expected due to demographic transition towards older age groups and increase in overweight and obesity among the population of Bangladesh. With more women being hypertensive than men, a targeted approach catering to high risk groups should be thoroughly implemented following the action points stated in the Multisectoral Action Plan 2018–2025 by the national NCDC program, i.e., bringing about dietary change, increasing opportunities for physical activity among adults; specially adult females focusing on goals to attain and continue normal BMI, mass awareness campaigns etc. Acting in close collaboration with other ministries/relevant sectors to bring an enabling environment for the citizens to modify their existing lifestyle to adopt healthy lifestyle choices is a prerequisite for adequate prevention. Carefully designed adult female centric interventions and interventions for older age group should be prioritized during the initial phases. While screening the adult population is essential, the public sector cannot possibly manage the ever-expanding numbers of hypertensives. The private sector and NGOs need to be drawn into the program to assist.

## Supplementary Information

**Additional file 1: Figure A1**. Predictive probabilities of hypertension prevalence by selected correlates.

**Additional file 2: Figure A2a & A2b**. Distribution of SBP and DBP among study population of 35 years or more.

## Data Availability

This study used data from Demographic and Health Surveys (DHS) for Bangladesh, which are available from the DHS programme website (www.dhsprogram.com). The data set can be found in the following link- https://www.dhsprogram.com/data/dataset/Bangladesh_Standard-DHS_2017.cfm?flag=0

## Abbreviations

BDHS: Bangladesh Demographic and Health Survey
BMMS: Bangladesh Maternal Mortality and Health Care Survey
BP: Blood pressure
BMI: Body mass index
DBP: Diastolic blood pressure
NCDs: Non-communicable diseases
NCDC: Non-communicable disease control
OCP: Oral contraceptive pill
STEPS: STEPwise approach to surveillance
SBP: Systolic blood pressure
UHC: Upazila Health Complex

## Footnotes

1 Streatfield PK, Iqbal A, Ahsan KZ, Khan SH, Haidar MH, Chakraborty N, Jamil K. Policy Brief: Hypertension and Diabetes in Bangladesh Based on Findings from BDHS 2017–18. Dhaka: NIPORT & icddr,b; 2020. [Forthcomimg]

2 Streatfield PK, Iqbal A, Ahsan KZ, Khan SH, Haidar MH, Chakraborty N, Jamil K. Policy Brief: Hypertension and Diabetes in Bangladesh Based on Findings from BDHS 2017–18. Dhaka: NIPORT & icddr,b; 2020. [Forthcoming]
