# Supplementary figures and images for: Demographic, socioeconomic, and biological correlates of hypertension in an adult population: evidence from the Bangladesh demographic and health survey 2017–18
Source: BMC Public Health. 2021 Jun 26;21:1229. doi: 10.1186/s12889-021-11234-5 (PMC8235611; doi:10.1186/s12889-021-11234-5)

Predictive margin with 95% CIs

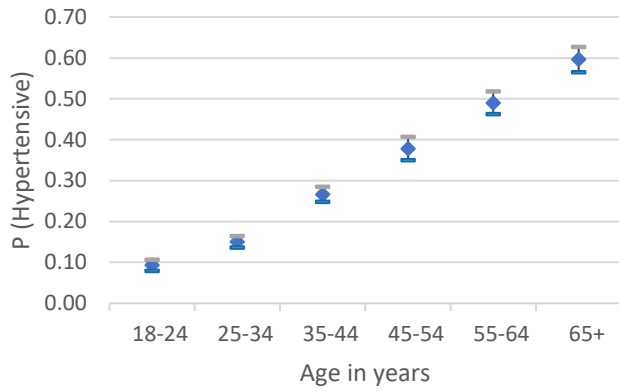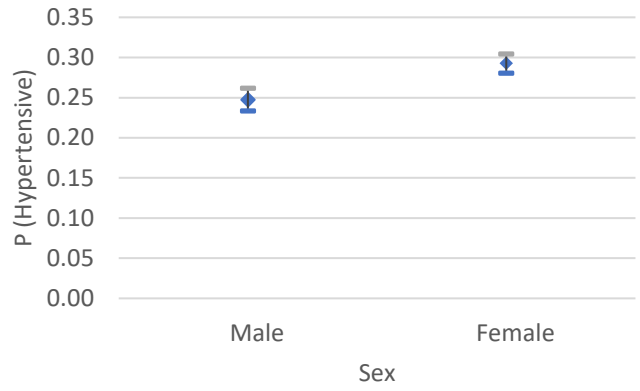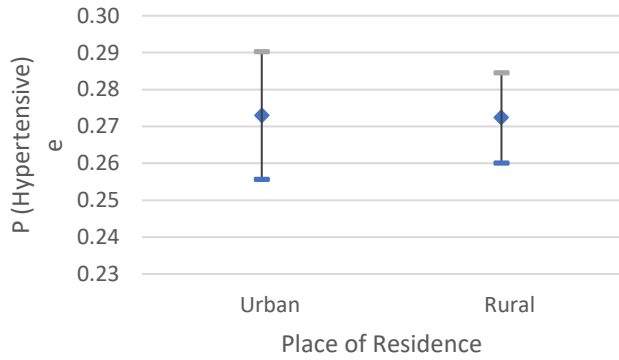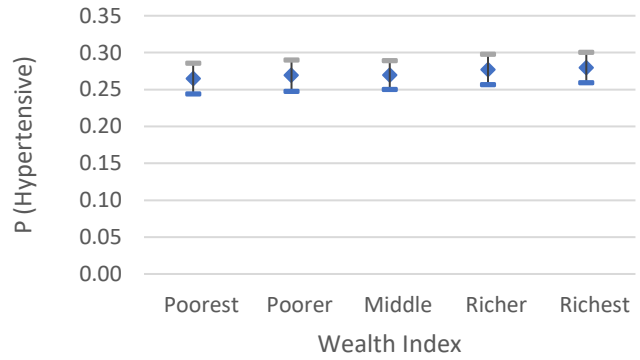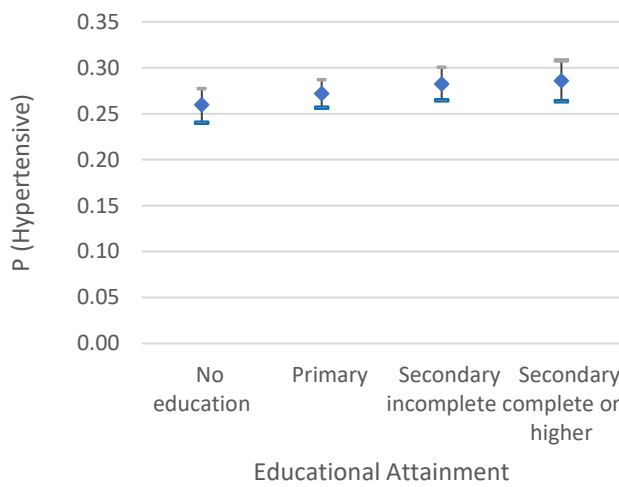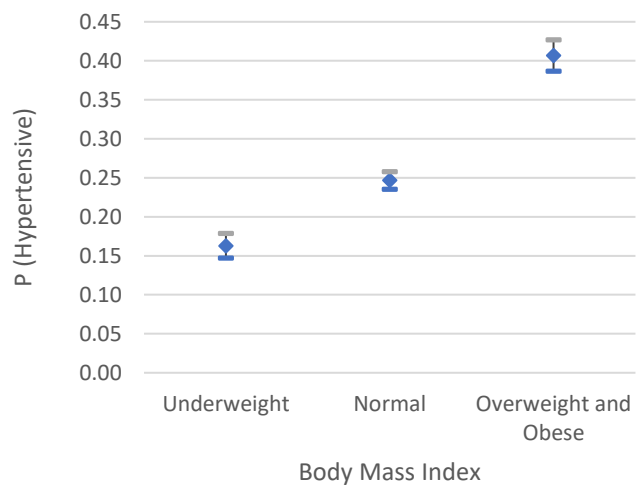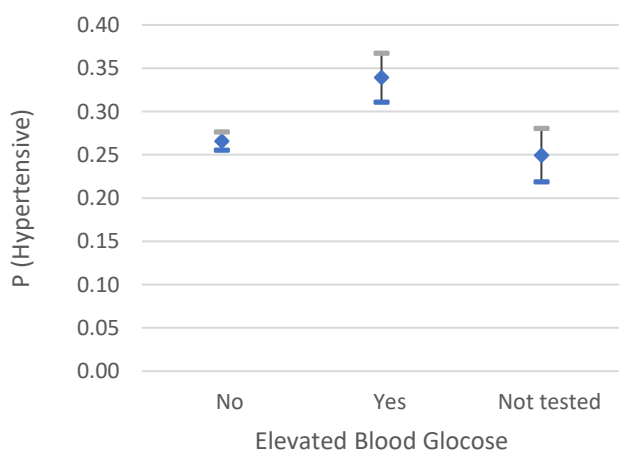

◆ Predictive margin  
— Lower CL  
— Upper CL

Supplement: Supplementary file 1 — Additional file 1: Figure A1. Predictive probabilities of hypertension prevalence by selected correlates. [file 12889_2021_11234_MOESM1_ESM.pdf]

SYSTOLIC BP, F+M, 2017 & 2011

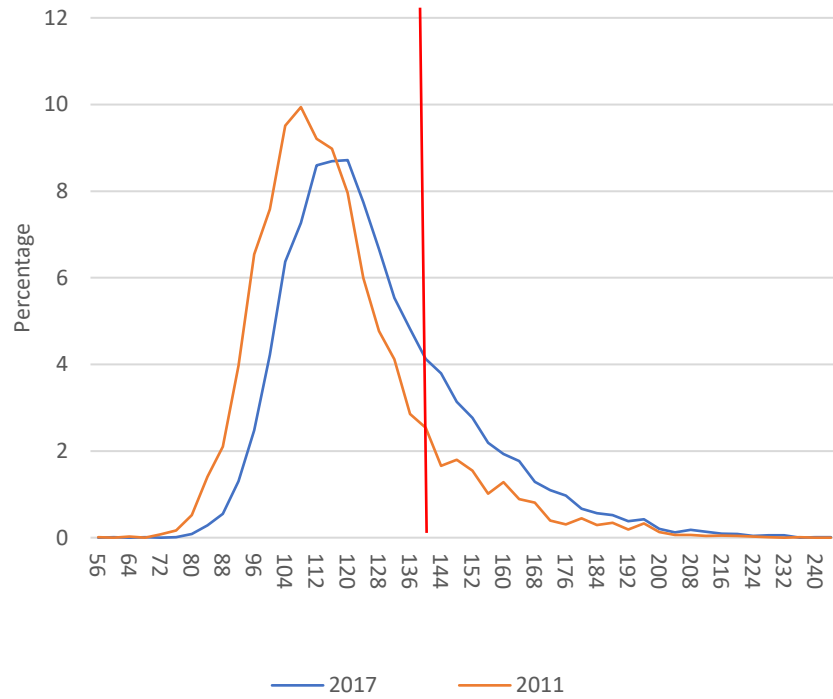

DIASTOLIC BP, F+M, 2017 & 2011

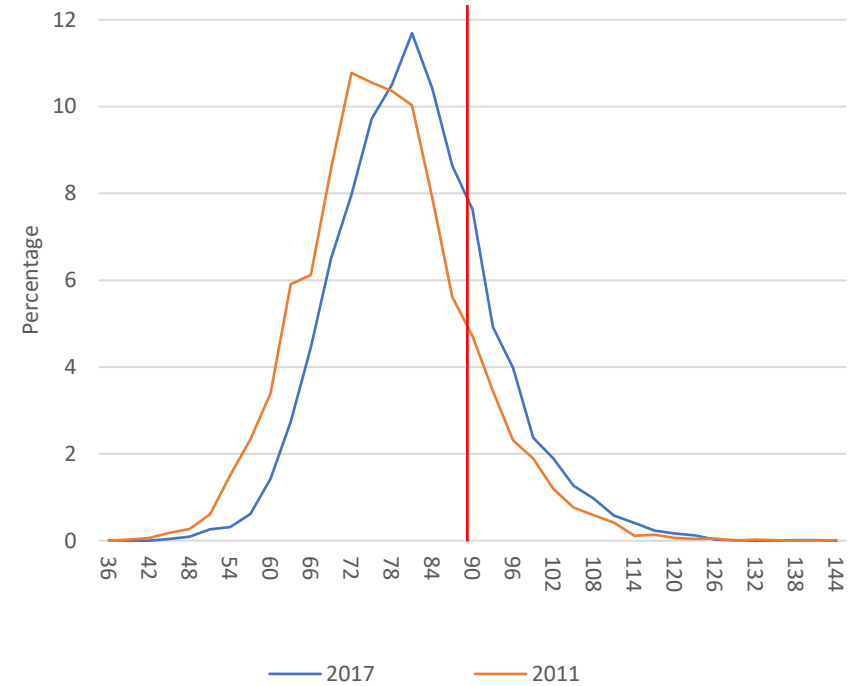

Supplement: Supplementary file 2 — Additional file 2: Figure A2a & A2b. Distribution of SBP and DBP among study population of 35 years or more. [file 12889_2021_11234_MOESM2_ESM.pdf]
